# Assessing the accuracy of low-cost optical particle sensors using a physics-based approach

**DOI:** 10.5194/amt-13-6343-2020

**Published:** 2020-11-26

**Authors:** David H. Hagan, Jesse H. Kroll

**Affiliations:** 1 Department of Civil and Environmental Engineering, Massachusetts Institute of Technology, Cambridge, MA 02139, USA; 2 QuantAQ, Inc., Somerville, MA 02143, USA; 3 Department of Chemical Engineering, Massachusetts Institute of Technology, Cambridge, MA 02139, USA

## Abstract

Low-cost sensors for measuring particulate matter (PM) offer the ability to understand human exposure to air pollution at spatiotemporal scales that have previously been impractical. However, such low-cost PM sensors tend to be poorly characterized, and their measurements of mass concentration can be subject to considerable error. Recent studies have investigated how individual factors can contribute to this error, but these studies are largely based on empirical comparisons and generally do not examine the role of multiple factors simultaneously. Here, we present a new physics-based framework and open-source software package (*opcsim*) for evaluating the ability of low-cost optical particle sensors (optical particle counters and nephelometers) to accurately characterize the size distribution and/or mass loading of aerosol particles. This framework, which uses Mie theory to calculate the response of a given sensor to a given particle population, is used to estimate the fractional error in mass loading for different sensor types given variations in relative humidity, aerosol optical properties, and the underlying particle size distribution. Results indicate that such error, which can be substantial, is dependent on the sensor technology (nephelometer vs. optical particle counter), the specific parameters of the individual sensor, and differences between the aerosol used to calibrate the sensor and the aerosol being measured. We conclude with a summary of likely sources of error for different sensor types, environmental conditions, and particle classes and offer general recommendations for the choice of calibrant under different measurement scenarios.

## 1 Introduction

Human exposure to aerosols is associated with adverse health impacts and increased mortality (Apte et al., 2018; Burnett et al., 2018; Cohen et al., 2017; Dockery et al., 1993). The source and composition of aerosols have been linked to a range of negative health impacts (Antonini et al., 2003; Hart et al., 2012; Henneberger and Attfield, 1997; Lipsett and Campleman, 1999), with more than 4 million annual deaths worldwide attributed to ambient particulate matter pollution (Cohen et al., 2017). Accurate estimates of aerosol sources and health impacts critically rely on measurements of particulate matter concentrations across indoor and outdoor environments worldwide.

In many countries, particulate matter (PM) pollution is regulated by national or local government agencies (e.g., the EPA in the United States) and is typically measured using federally approved reference methods that are high in accuracy and precision. The existing infrastructure is generally designed to measure regional-scale air pollution in order to enforce (and assess the effectiveness of) air quality regulations. However, particle pollution can vary in space and time at much finer resolution than can be measured using standard monitoring technologies given their relatively high cost and size. Over the past several years, new technologies have emerged at price points (< USD 2000) that allow PM measurements to be made with much higher spatiotemporal resolution, even down to the individual human level (Koehler et al., 2019; Tryner et al., 2019a, b). These devices are physically small, use very little power, and can easily be deployed at scale. As a result, such sensors are ideally suited for use in dense distributed sensor networks, providing high-resolution air quality measurements, as well in as personal monitoring, providing individuals with the ability to measure and understand their exposure to harmful air pollutants. As with all low-cost sensors (LCSs), accuracy is of paramount concern; as shown by a number of recent laboratory and field-based evaluation studies (Crilley et al., 2018; Dacunto et al., 2015; Di Antonio et al., 2018; Holstius et al., 2014; Levy Zamora et al., 2019; Malings et al., 2020; Northcross et al., 2013; Sousan et al., 2016b, a; Wang et al., 2015), PM sensors can perform quite poorly without additional constraints or calibrations.

Most low-cost PM sensors measure particles via light scattering. Sampled particles intercept a beam of light (typically from a laser or LED with a wavelength between 405 and 780 nm), and the scattered light is measured and correlated with a PM mass concentration. In this work, we refer to such instruments as optical particle sensors (OPSs). OPSs can be broken down into two main types, nephelometers and optical particle counters (OPCs). Nephelometers measure the particles as an ensemble, gathering light scattered by all particles across a wide range of angles, typically 7–173° to avoid pure forward and backward scattering (Abu-Rahmah et al., 2006; Ahlquist and Charlson, 1967; Anderson et al., 1996). The total scattering amplitude is then correlated with a mass measurement made by a reference instrument. (Nephelometers that measure scattered light at a single angle are sometimes referred to as photometers; for the purposes of this work we consider photometers to be a subclass of nephelometer.) OPCs, by contrast, detect particles individually, providing information on their number and size. Light scattered by each individual particle is measured and each pulse is assigned to a size bin based on its total light intensity, resulting in a histogram which is converted to a mass loading once the entire distribution has been measured. While these technologies have been around for decades (Gucker et al., 1947; Patterson et al., 1926), they have recently become available at much lower cost due to the availability of small, inexpensive light sources and electronic components.

The use of light scattering introduces a number of fundamental limitations for making PM mass measurements. Many of these arise from environmental conditions and/or the properties of the aerosol being measured; these can be especially problematic when calibration is done using only a single aerosol type or condition. A number of recent empirical studies of OPSs have investigated some of these limitations. These issues include (1) the inability to adapt to changes in the particle size distribution (Dacunto et al., 2015; Wang et al., 2015), (2) the hygroscopic growth of particles due to changes in ambient relative humidity (Crilley et al., 2018; Di Antonio et al., 2018; Malings et al., 2020; Zheng et al., 2018), (3) changes in scattering efficiency due to differences in aerosol optical properties (Crilley et al., 2018; Di Antonio et al., 2018), and (4) the need for aerosol-specific correction factors to account for differences in density (Dacunto et al., 2015; Northcross et al., 2013). While these studies have examined how these individual effects in isolation may affect PM accuracy, to our knowledge there has not been a systematic, comprehensive investigation of all these factors together. Complicating matters is the fact that these individual properties are all intertwined - for example, when relative humidity increases, it can cause particles to take up water, which can change not only their size and mass but also their shape, refractive index, and density.

To examine the relative contribution of error by various interacting sources, we have developed a model that describes how a given sensor will respond to different aerosols under a wide range of conditions. This model is based entirely on the underlying physics of light scattering (Mie theory) rather than empirical relationships obtained through laboratory or field measurements. While previous work has modeled nephelometers and OPCs in a similar way (Walser et al., 2017), we believe this is the first detailed treatment of light scattering as it relates specifically to LCSs. We use this model to isolate the relevant sources of error and develop a better understanding of the limitations (as well as strengths) of different kinds of OPSs.

The modeling tool described here, which is open-source and freely available, can be used for the systematic study of how different OPSs may detect various aerosol types under a range of environmental conditions. This enables new insights into the potential errors associated with a given PM measurement, optimal strategies for calibrating OPSs, and ultimately the design of the sensors themselves and the development of algorithms for data analysis. The objective of this work is to describe the model and software and to investigate broad influences of aerosol properties and sensor parameters on measurement performance. This present work does not investigate the performance of individual commercially available sensors under the full range of conditions expected in the atmosphere, but such studies are enabled by this modeling tool and are an important future extension of this work.

## 2 Methods

The modeling framework described in this section is available as an open-source (MIT license) Python library (*opcsim*) and has been made available on GitHub. Detailed documentation, including installation instructions and examples, is available online (Hagan and Kroll, 2019). The framework, called “opcsim”, consists of two primary components: the code that models OPSs and implements the Mie theory algorithms (Bohren and Huffman, 1983; Sumlin et al., 2018) and the code to build and evaluate aerosol distributions.

We follow the same general modeling pattern regardless of sensor type. Steps include (1) defining the device based on its key physical parameters, (2) calibrating the device to a specific aerosol type (for OPCs) or aerosol distribution (for nephelometers), and (3) evaluating each particle in an aerosol population by computing the scattered light signal using Mie theory and converting that signal to the sensor output based on its calibration. In the following sections we describe how the aerosol population is described by the model, followed by how the sensors themselves are treated.

### 2.1 Representing an aerosol distribution

We represent an aerosol distribution as the sum of *n* lognormal modes, whereby each mode *i* is defined by its geometric mean particle diameter (${\bar{D}}_{\text{p},i}$), geometric standard deviation (σ_*i*_), and number concentration (*N*_*i*_). The aerosol distribution as a function of diameter *D*_p_ (d*N/*dlog *D*_p_) is given by Eq. (1) (Seinfeld and Pandis, 2006):

(1) $$\frac{\text{d}N}{\text{dlog}D_{\text{p}}}=\sum_{i=1}^{n}\frac{N_{i}}{\sqrt{2\pi }\text{log}\sigma_{i}}\text{exp}\left(-\frac{{\left(\text{log}D_{\text{p}}-\text{log}{\bar{D}}_{\text{p},i}\right)}^{2}}{2{\text{log}}^{2}\sigma_{i}}\right).$$

Additionally, we define the composition of the aerosol distribution by defining the particle density (*ρ*_*i*_), hygroscopic growth factor (*κ*_*i*_), and complex refractive index (*m*_*i*_) for each mode. The role of these additional parameters is discussed in Sect. 3 below. While more complex representations of the chemical makeup of the aerosol can be implemented using our modeling framework (i.e., core-shell representation of aerosols, complex aerosol mixtures), for the purposes of this paper we focus only on well-mixed homogeneous particle modes, as described by Eq. (1). The above number distribution can be converted to a mass distribution (or total mass concentration) by assuming all particles are spherical with a known density (Seinfeld and Pandis, 2006).

### 2.2 Representing optical particle sensors

#### 2.2.1 Optical particle counters (OPCs)

An OPC is defined by three instrument-specific parameters: (1) the wavelength of the light source (λ), (2) the viewing angle for which the scattered light is collected, and (3) the number of discrete size bins and their widths. A bin, in this context, refers to a single “slice” of the aerosol size distribution, with a fixed width and units of particle diameter. Typically, most low-cost OPCs have between 2 and 30 bins. These can be determined either by looking up the parameters in the device’s data sheet provided by the manufacturer or by making simple measurements. Bins are often chosen to reduce the uncertainty in correct bin assignments within the bounds of what the sensor is capable of detecting. Most low-cost OPCs have the smallest bin at *D*_min_ ~ 500 nm, with cost typically being the driving factor – OPCs with lower *D*_min_ employ more expensive, higher-quality optics and photodetectors, allowing them to accurately detect smaller particles. In this work, the bin boundaries (and hence widths) used for a given OPC are taken from the manufacturer’s spec sheets if available; otherwise, they are calculated by generating an array of logarithmically spaced bin boundaries for a set number of bins (*n*_bins_) between the minimum and maximum defined diameters (*D*_min_ and *D*_max_, respectively). Most often, a light pulse generated by a single particle is assigned to exactly one bin. However, there are approaches whereby bin assignments are made using a probability distribution (Walser et al., 2017); this is not implemented in this model but is an approach that could be added in the future. Table 1 lists bin widths and other parameters for a few commercially available low-cost OPCs.

OPCs are calibrated by relating the scattered light intensity - a combination of the particle’s scattering cross section (*C*_scat_) and laser intensity - to the particle diameter. Practically, this is done by using calibration aerosols with known optical properties and size and generating a calibration curve between the test aerosol and the electronic pulse height generated by that aerosol. After repeating this process for many sizes, a calibration curve can be generated. Here, we compute the *C*_scat_ values with Mie theory using attributes of the calibration aerosol. To simplify the model, we make several assumptions, including the following: (1) all particles are spherical and homogeneous (well-mixed); (2) the laser intensity is constant, implying all particles are perfectly centered in the beam of the laser; and (3) the photodetector and electronics are 100 % efficient, so we do not consider the impact of signal-to-noise limitations.

As most low-cost OPCs contain an elliptical refocusing mirror to gather the scattered light across many angles, we compute the integrated light-scattering intensity following a procedure first introduced by Jaenicke and Hanusch (Jaenicke and Hanusch, 1993). Mie theory calculations are implemented using equations by Bohren and Huffman (Bohren and Huffman, 1983). The scattering cross section is calculated as

(2) $$C_{\text{scat}}=\frac{\lambda }{4\pi }\int_{\Theta_{1}}^{\Theta_{2}}\left[i_{1}(\Theta )+i_{2}(\Theta )\right]\text{sin}\Theta \text{d}\Theta ,$$

where λ is the wavelength of incident light, Θ is the viewing angle (which ranges from Θ_1_ to Θ_2_), and *i*_1_ and *i*_2_ are the intensity distribution functions (Bohren and Huffman, 1983).

**Figure 1.**
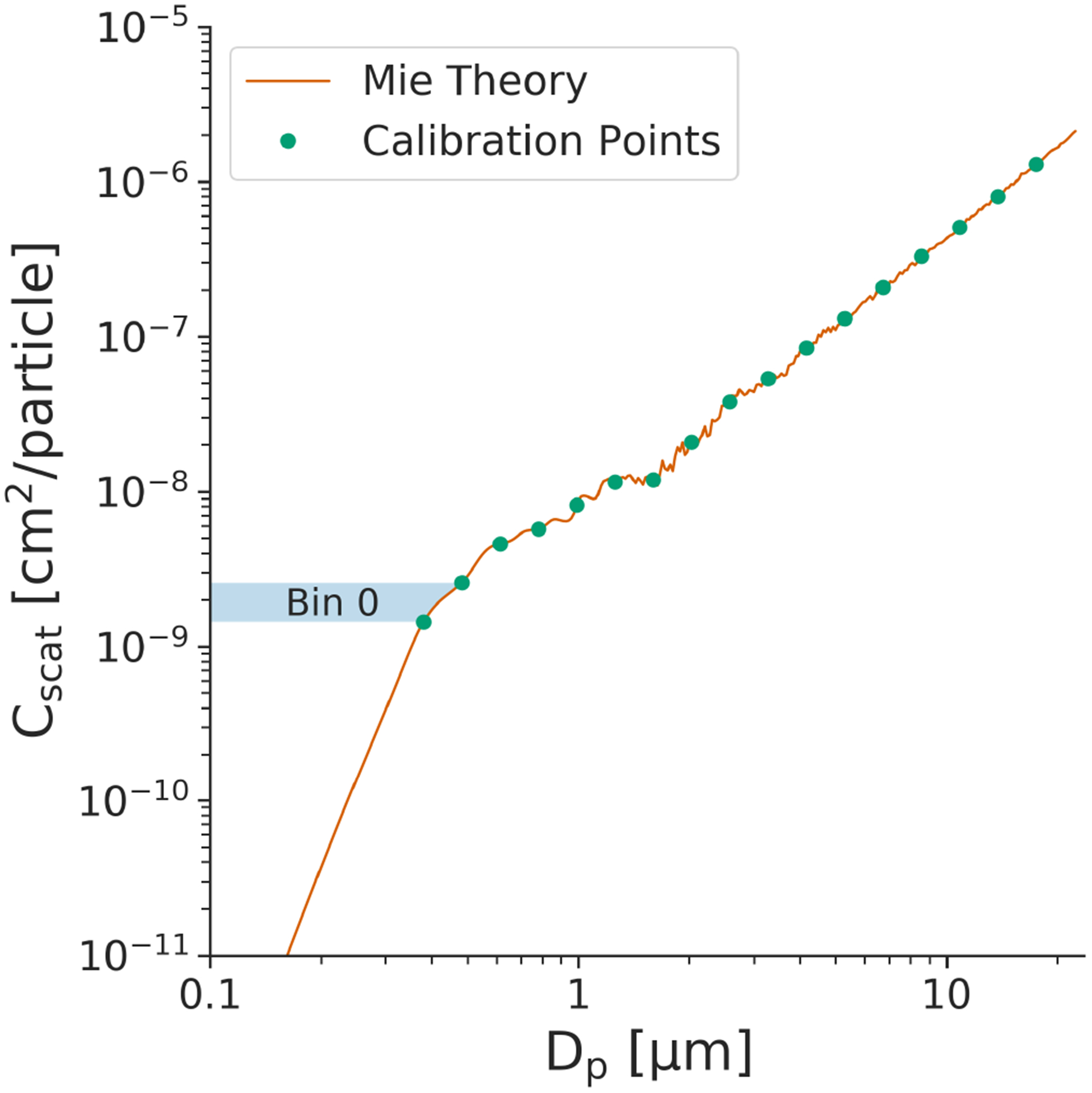
Calibration data for an OPC with 16 discrete size bins between 0.38 and 17.5 μm. OPC parameters were chosen to match the Alphasense OPC-N2 (wavelength of 658 nm, viewing angle of 32–88°) using monodispersed polystyrene latex spheres (*m* = 1.592 + 0*j*). The integrated scattering amplitude calculated using Mie theory is shown as the solid line, with points depicting the corresponding scattering amplitude at each of the bin boundaries. Shown as a shaded box is the range of scattering amplitudes that is assigned to the smallest size bin.

Figure 1 depicts the calibration curve generated for an OPC with the characteristics of the Alphasense OPC-N2 (Table 1) using polystyrene latex spheres (PSLs) of different diameters for calibration. Equation (2) was used to compute the theoretical *C*_scat_ values (*y* axis) integrated across the entire viewing angle for a range of particle diameters (*x* axis). The *C*_scat_ values at each bin boundary (green dots in Fig. 1) are then computed, and spline interpolation is used between each individual bin boundary to generate a mapping between the scattering amplitude and its corresponding bin assignment. In practice, this operates as a lookup table - a particle crossing the laser generates a scattering amplitude that is associated with a specific “bin” via the calibration.

For OPCs that measure scattered light across a wide angle, *C*_scat_ is generally a monotonically increasing function of the particle size. However, there may be cases in which this is not true, typically due to the presence of Mie resonance (e.g., near *D*_p_ = 1.5 μm in Fig. 1). When the function is not monotonic, we apply a smoothing algorithm (Cerni, 1983; Osborne et al., 2008) or merge together multiple bins (Pinnick et al., 1981; Walser et al., 2017) and accept the trade-off whereby we obtain a higher rate of correct bin assignment in exchange for reduced bin resolution. This non-monotonicity is less of an issue as the viewing angle becomes wider, as the larger range of angles will “smooth out” any Mie resonances (Fig. S1 in the Supplement). The wide viewing angle thus offers two key advantages: (1) the total signal (pulse height) is larger, making it easier to detect small particles using inexpensive electronics, and (2) the calibration curve is less susceptible to small changes in the particle scattering cross section.

While an OPC sizes and counts individual particles, we are generally interested in evaluating the entire population of particles. To obtain the results for the entire population, we compute the scattering cross section for each particle in the distribution and assign it to a bin using the calibration curve generated previously - this results in a histogram with the total sum of particles in each discrete size bin over a period of time. Once we have the number distribution, we can compute the aerosol mass loading (PM) using Eq. (3):

(3) $$\text{PM}=\rho \sum_{i}N_{i}\frac{\pi }{6}D_{\text{p},i}^{3},$$

where *N*_*i*_ is the number concentration for a given size bin, *D*_p*,i*_ is the geometric mean diameter for a given size bin, and *ρ* is the particle density, chosen to be constant. We can integrate mass loadings between different diameters by summing only across a sub-selection of bins (for example, if we intend to calculate the PM_1_ mass concentration, we would choose only the size bins corresponding to particles sized between 0 and 1 μm, whereas to calculate the PM_2.5_ mass concentration, we would use the bins corresponding to sizes between 0 and 2.5 μm). This approach for computing mass loadings is similar to that used by others (Di Antonio et al., 2018), though we use the geometric mean particle diameter as opposed to the mean particle diameter.

#### 2.2.2 Integrating nephelometers

Nephelometers gather the light scattered by an aerosol population across a wide range of angles to gather as much of the scattered light as possible, while avoiding the near-forward and near-backward scattered light. Here, we define a nephelometer by the wavelength of its light source (λ) and its viewing angle.

In practice, nephelometers are empirically calibrated by correlating the total scattered light signal with a reference mass measurement (Dacunto et al., 2015; Sousan et al., 2016b; Wang et al., 2015). Within our model, we do the same by computing the total scattered light signal using Mie theory and then take the ratio of the scattered light to a calculated mass loading. The total scattered light signal is calculated by integrating Eq. (2) across the entire particle size distribution, resulting in a single scattered light intensity for a given aerosol distribution. The calibration factor is then calculated by taking the ratio of this value and the mass loading of the aerosol distribution, which is calculated by integrating the volume distribution and multiplying by the particle density (Eq. 3). Once we have computed the calibration factor, we can calculate the mass loading of the measured aerosol distribution by multiplying the calibration factor by the calculated total scattered light signal.

## 3 Results and discussion

We use the model described above to isolate the relative sources of error associated with various differences in physical and optical properties of aerosols as well as with the devices themselves. We include both simple, targeted experiments probing the effects of changes in isolated properties and more complex, realistic experiments that attempt to mimic real-world scenarios. In the latter case, we include a variety of aerosol types in our model runs to resemble real-world use cases; aerosol types include urban aerosol, wildfire emissions, marine aerosol, dust, and continental background. The physical and optical properties for these aerosols are summarized in Table 2. We discuss these results in the context of three particle sensors chosen to be representative of low-cost OPSs: a nephelometer, which uses a 658 nm light source and has a viewing range of 7–173°, and two OPCs, both with 16 equally spaced bins, a 658 nm light source, and a viewing angle of 32–88°. The two OPCs differ only in the minimum particle size measured: the “low-cost OPC” is representative of commercial OPCs currently on the market and measures particles in the 0.38–17.5 μm size range, and the “high-end OPC” represents an idealized OPC that can measure much smaller particles with a detection range of 0.1–17.5 μm. We note that many expensive OPCs cannot measure particles down to 100 nm; this lower size cutoff was chosen as an approximate smallest particle size that an optical sensor can detect.

We begin by investigating the impact that water uptake, driven by changes in the ambient relative humidity, has on the ability of all three OPSs to infer PM_2.5_ mass. Next, we explore the impact of aerosol optical properties (namely, the complex RI), followed by the impact that perturbations in the underlying particle size distribution can have on OPS ability to infer mass loadings. Finally, we summarize our results into general recommendations about each OPS type. Throughout, to provide a simple metric for the accuracy of OPS measurements, we present our results in terms of the ratio of the inferred or measured PM_2.5_ mass concentration (*M*_m_) to the actual PM_2.5_ mass concentration (*M*_a_) at 0 % relative humidity. An *M*_m_*/M*_a_ ratio greater than 1 implies we are overestimating the PM_2.5_ loading, whereas a value less than 1 implies we are underestimating it.

### 3.1 Relative humidity and hygroscopic growth

One of the most widely discussed sources of error for OPS measurements is that caused by water uptake (Crilley et al., 2018; Di Antonio et al., 2018; Malings et al., 2020; Wang et al., 2015; Zheng et al., 2018). As relative humidity increases, hygroscopic particles (those with nonzero hygroscopic growth parameters, *κ*) become larger as they take up water (Petters and Kreidenweis, 2007), leading to an increase in scattering caused by their increase in size. Additionally, water uptake changes the optical and chemical properties of the aerosol (e.g., RI, density), which can complicate any corrections. The EPA requires PM_2.5_ measurements to be made at relative humidities between 30 % and 40 % (Chow and Watson, 1998) to minimize the effects of hygroscopic growth on samples; however, since very few low-cost OPSs control for relative humidity (for example, with an in-line dryer), this can often lead to errors when performing a calibration by co-location or when comparing results between instrument types.

**Figure 2.**
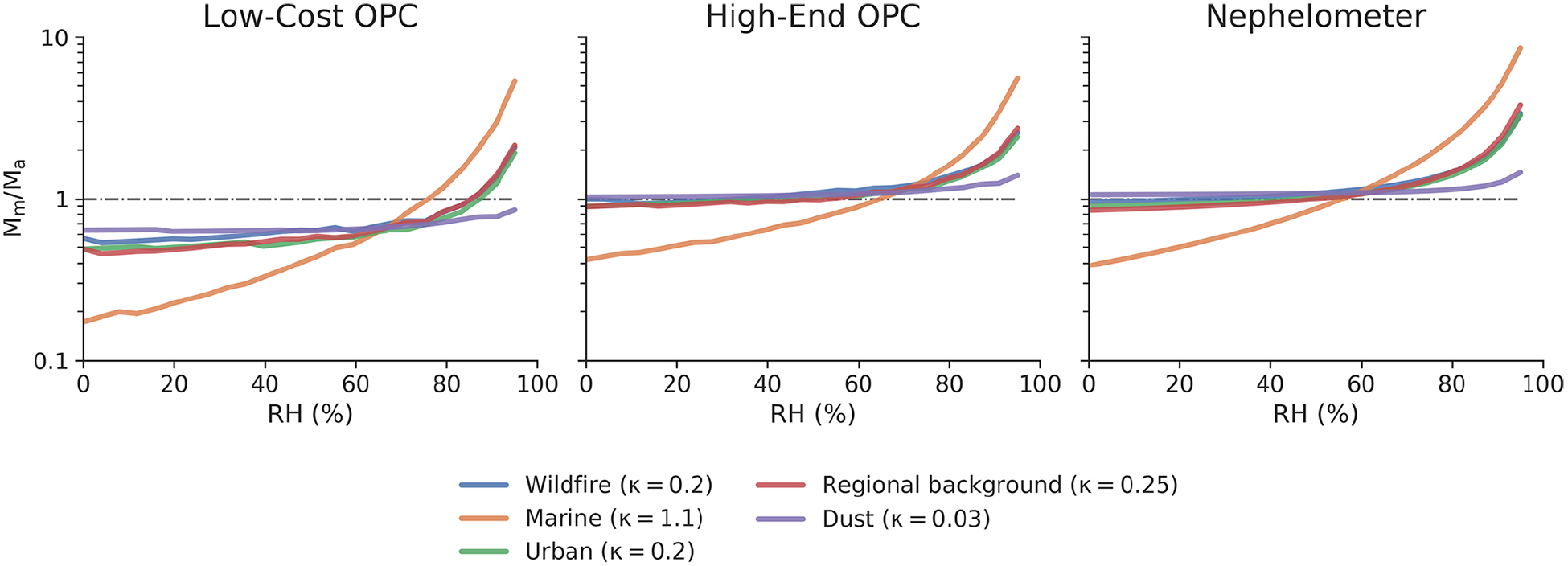
The accuracy in PM_2.5_ mass loading for a given particle sensor (*M*_m_*/M*_a_) as a function of relative humidity for common aerosol types. All three particle sensors were calibrated with ammonium sulfate (number-weighted geometric mean (GM) = 200 nm, geometric standard deviation (GSD) = 1.65). Details on the physical and optical properties of the various aerosols can be found in Table 2.

Figure 2 shows the impact that RH can have on the accuracy of an OPS. There is little effect until relative humidity reaches the deliquescence point of the aerosol, which depends on aerosol composition. At higher relative humidities, OPSs will tend to overestimate PM_2.5_ mass, especially for aerosols comprised of hygroscopic materials. When relative humidity approaches 95 %, such overestimates in PM_2.5_ mass become exceedingly large: the OPCs observe a similar effect, with errors ranging 100 %–500 % depending on the hygroscopicity of the aerosol. Nephelometers see a more pronounced effect, with errors as high as 750 % for extremely hygroscopic aerosols and 200 %–300 % for less hygroscopic aerosols.

The larger error of the nephelometer is caused in part by the fact that the PM_2.5_ mass is directly proportional to the total scattered light, which has no upper limit. For the OPCs, particles that take up significant water can be assigned to larger size bins and thus will not be integrated in the PM_2.5_ mass calculation. At moderate humidities (50 %–80 %), errors for both the nephelometers and OPCs can vary by as much as 20 %–50 %, which is in agreement with a number of published experimental studies on the subject (Crilley et al., 2018; Di Antonio et al., 2018; Malings et al., 2020; Zheng et al., 2018). In addition to overestimating mass loadings at high relative humidity due to hygroscopic growth, the OPCs underestimate the mass loadings across all relative humidities. This is not caused by relative humidity or a lack of hygroscopic growth but is instead a result of the “missing mass” below the detectable threshold of the OPC. The low-cost OPC, which cannot detect particles smaller than 380 nm, misses between 30 % and 90 % of the mass, whereas the high-end OPC, which can detect particles larger than 100 nm, misses very little mass for most aerosol types. The only exception is marine aerosol, which has a refractive index that is substantially different from the aerosol with which the instrument was calibrated.

### 3.2 Choice of calibration material and aerosol optical properties

**Figure 3.**
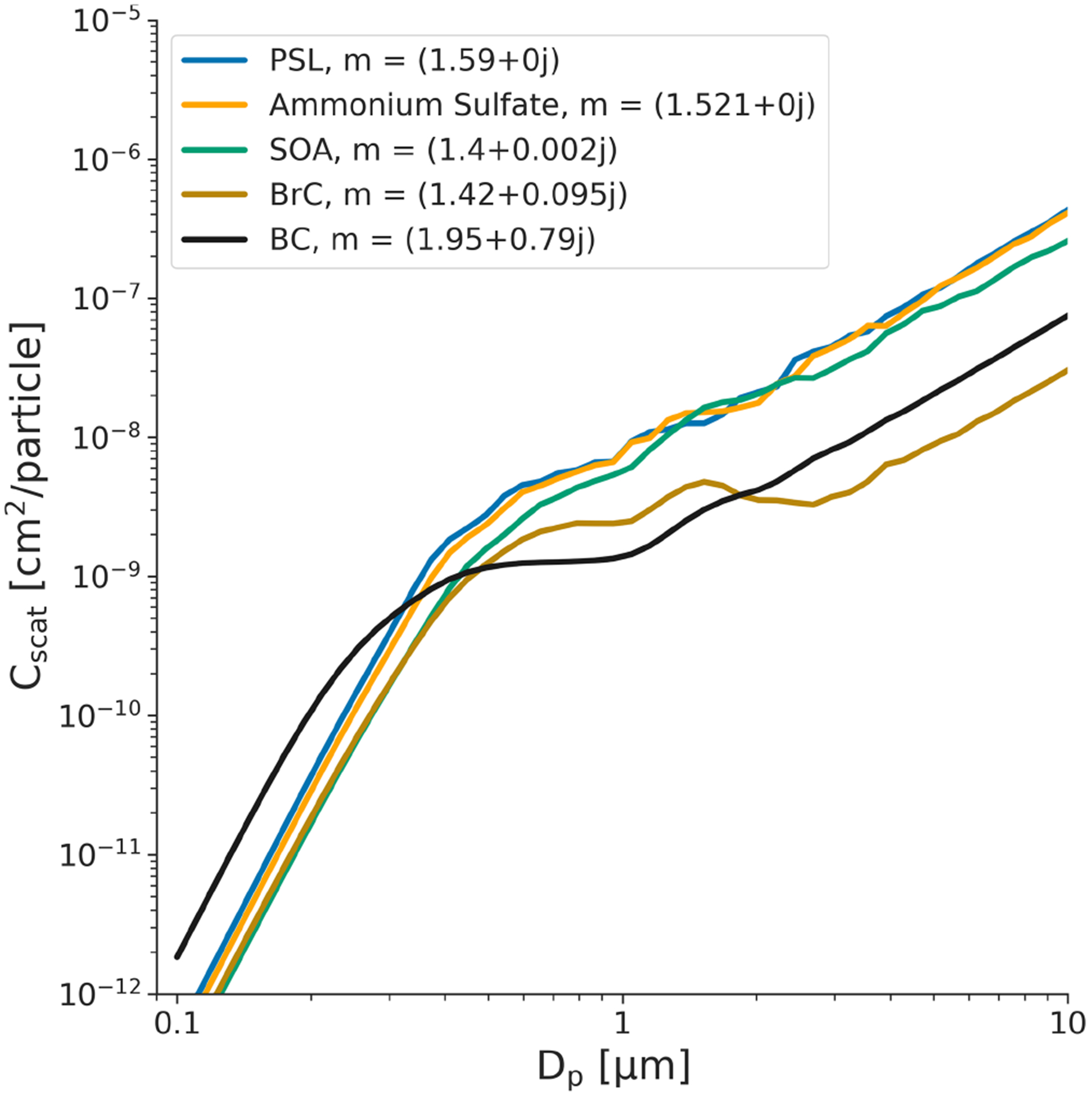
Mie curves (integrated over a viewing angle of 32–88°) for a select group of common calibration materials. Materials shown include polystyrene latex spheres (PSLs), ammonium sulfate, secondary organic aerosol (SOA), and black carbon (BC). Small differences in the refractive index of a measured material can lead to drastic bin misassignment depending on where bin boundaries are set at the time of calibration.

OPCs are calibrated by correlating the scattering amplitude of known particle sizes for particles of a given composition (Gao et al., 2013). The relationship between scattering amplitude and bin assignment (i.e., particle size) is heavily dependent on the aerosol’s complex refractive index (RI). Figure 3 shows the Mie scattering curve for a range of common calibration materials, including both absorbing and non-absorbing materials. For a given particle size, the RI of the particle can result in a range of scattered light intensities (*C*_scat_) that vary by as much as an order of magnitude. This can have pronounced effects on the calculated size (and hence mass) of a particle. In particular, the Mie curve for black carbon (BC) is substantially different from that of non-absorbing materials. As a result, for an OPC calibrated with a non-absorbing material (such as PSLs), smaller BC particles (diameters < 300 nm) will be overestimated in size, whereas larger BC particles (> 300 nm) will be underestimated. Even small changes in the scattering (real) component of the RI of the calibration material can lead to particles being assigned to the incorrect bin: an RI higher than that of the calibration material will generally cause particles to be assigned to bins that are too large (overestimating size and mass), and an RI lower than that of the calibration material will generally cause particles to be assigned to bins that are too small (underestimating size and mass). Considering that bins are often at least hundreds of nanometers in width, the impact of such bin misassignment on reported mass can be large. For both OPCs and nephelometers, this will lead to large errors in inferred mass, though it can be more pronounced for OPCs, since the error for nephelometers is proportional to the increase in scattering and is not affected by the misassignment of individual particles to a particular size bin.

**Figure 4.**
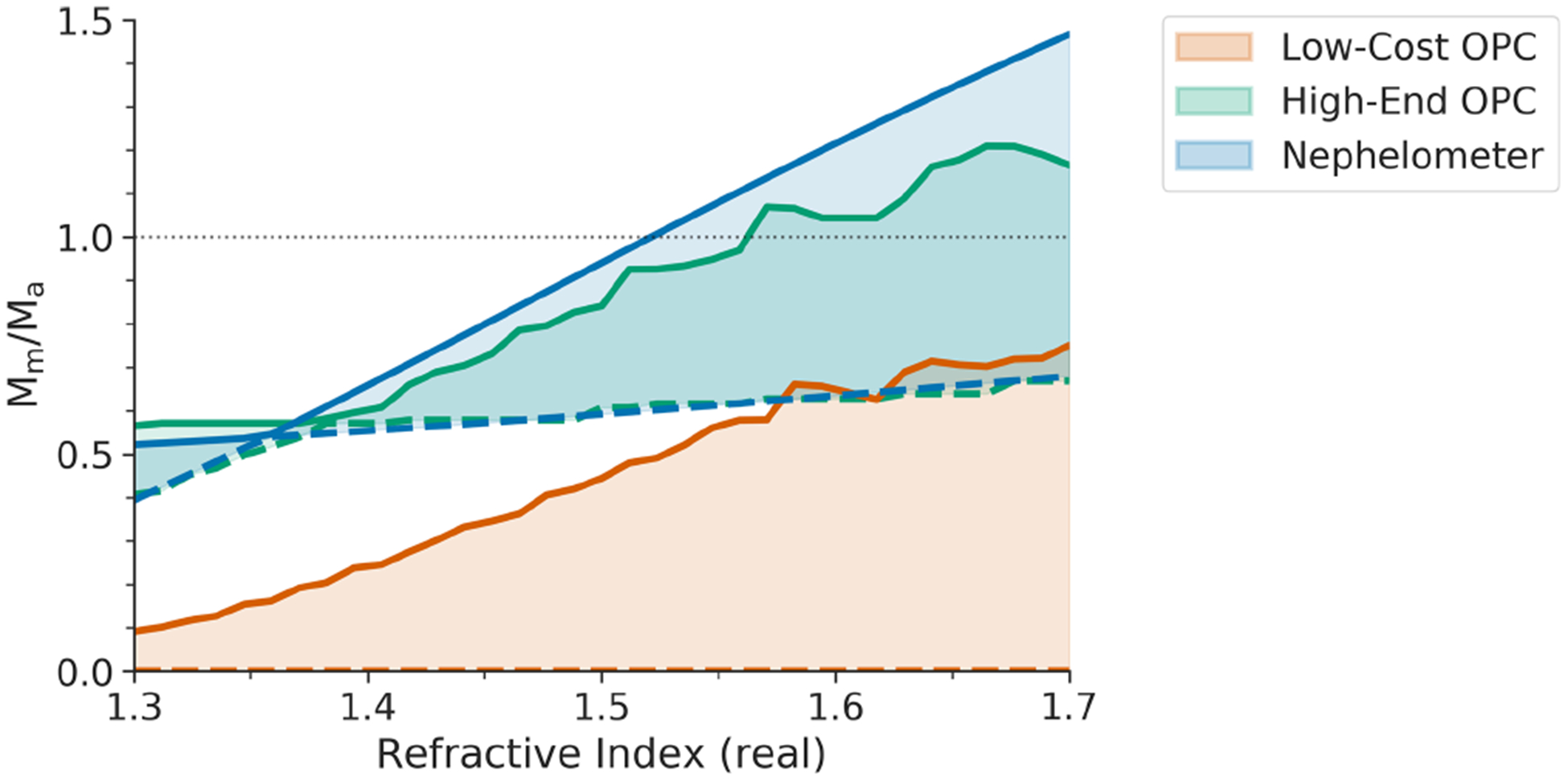
The accuracy of OPSs as a function of the refractive index of the aerosol being measured. The real component of the RI is on the *x* axis, and the width of each swatch is bounded by the absorption and imaginary component, which spans from 0 (non-absorbing, solid line) to 0.79 (black carbon, dashed line). Results are shown for a nephelometer (blue) and the two OPCs (orange and green). All results are for a generic particle size distribution with number-weighted GM = 200 nm and GSD = 1.65, and the OPSs were calibrated with PSLs.

The effect of differences in refractive index on inferred PM_2.5_ mass measurements is shown in Fig. 4. Results are shown for a single aerosol distribution, in which the only parameter allowed to vary is the RI. The real component of the refractive index is shown on the *x* axis, with the upper and lower bounds being determined by the imaginary part of the refractive index; the imaginary component ranges from 0 (non-absorbing) to 0.79 (black carbon). The nephelometer (blue swatch in Fig. 4) is calibrated using PSLs (*m* = 1.59 + 0*j*). When the nephelometer is evaluated at this exact RI (and a constant size distribution), it measures mass accurately (*M*_m_*/M*_a_ = 1). However, if the real component of the aerosol being evaluated is higher than that of the calibration standard, the total scattering is greater, resulting in the inferred PM_2.5_ mass being larger than the actual PM_2.5_ mass (*M*_m_*/M*_a_ > 1). Similarly, as the absorbing component becomes larger, less of the incoming light is scattered, resulting in a substantial underestimation of the mass loading.

Also shown are the results for two OPCs. The high-end OPC (green) is sensitive to particles as small as 100 nm, whereas the low-cost OPC (red) is sensitive to particles as small as 380 nm. As the absorbing component of the refractive index becomes larger, the scattering amplitude across the entire distribution is too small for the OPC to detect, resulting in a mass reading of zero. Both OPCs exhibit this effect, but for the high-end OPC, fewer particles will fall below the size cutoff of the OPC than for the low-cost OPC, resulting in a less dramatic underestimate of the mass. Most commercially available OPCs are more similar to the low-cost OPC, with *D*_min_ of around 500 nm. If operating in an environment where the aerosol is strongly absorbing, large underestimates in PM_2.5_ should be expected. Even under conditions in which the aerosol is not absorbing, the low-cost OPC largely underestimates the mass due to its high minimum size cutoff. For nephelometers, the errors are not as drastic but still strongly depend on the RI of the calibration aerosol used.

### 3.3 Changes in the particle size distribution (PSD)

**Figure 5.**
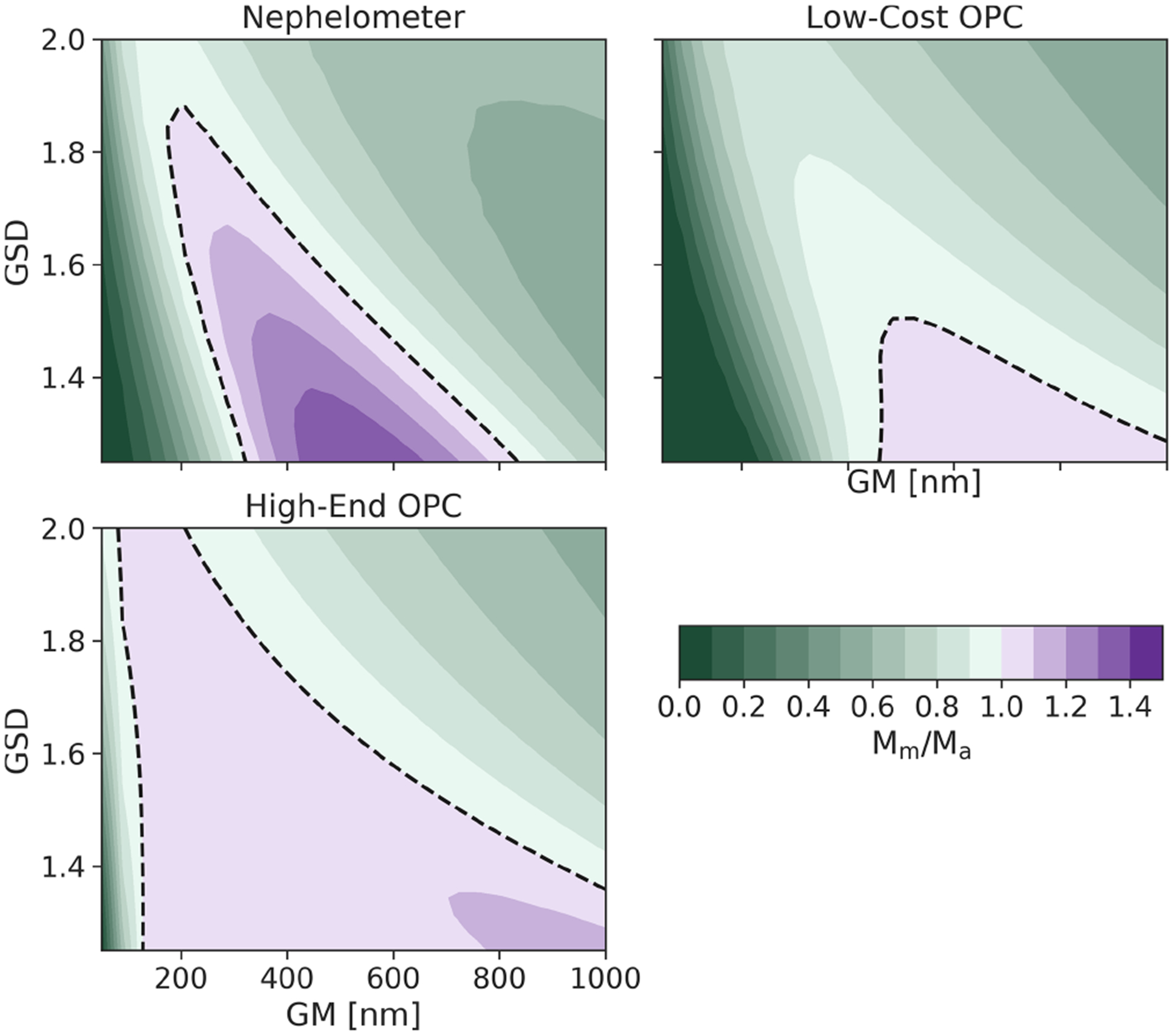
Mass concentration accuracy (*M*_m_*/M*_a_) of OPSs for a range of particle size distributions (PSDs). Accuracy is shown for all combinations of PSDs with number-weighted geometric mean diameters (GMs) between 100 and 1000 nm and geometric standard deviations (GSDs) between 1.2 and 2.0. Perturbations in the PSD can lead to large errors for nephelometers and optical particle counters with high minimum particle size cutoffs. All results are shown for ammonium sulfate particles; the OPCs were calibrated with PSLs, and the nephelometer was calibrated with ammonium sulfate (*N* = 1 × 10^4^ cm^−3^, GM = 400 nm, and GSD = 1.5). A black dashed line indicates the 1 : 1 line, where *M*_m_*/M*_a_ = 1.

The ability of optical particle sensors to adapt to perturbations in the underlying particle size distribution (PSD) is important because PSDs can be highly variable over short periods of time, especially in urban areas with highly varying contributions from various local sources. Figure 5 shows the accuracy of all three OPSs as the function of the PSD of the particles being measured. These calculations assume a single lognormal mode with all other properties of the aerosols (density, refractive index, and hygroscopicity) held constant. For the purpose of the model, the OPCs were calibrated using PSLs at each bin boundary, and the nephelometer was calibrated using ammonium sulfate (*N* = 1 × 10^4^ cm^−3^, GM = 400 nm, and GSD = 1.65). The entire population of ammonium sulfate particles is then evaluated while varying the number-weighted geometric mean particle diameter (GM) and the geometric width of the distribution (GSD). For each PSD, we compute the relative accuracy of each device and plot the results in Fig. 5, in which the color and contours correspond to the *M*_m_*/M*_a_ metric.

The nephelometer substantially underestimates the mass concentration (by 50 %–70 %) for most PSDs, since it is calibrated to a single PSD. As the PSD changes, the ratio of total scattered light to integrated mass changes, causing the accuracy to change as well. OPCs are potentially better since they measure the size of the particles and can theoretically account for changes in the PSD; however, they are still subject to errors given their limitations in detected size range. In particular, the low-cost OPC considerably underestimates the mass (by 60 %–90 %) for most PSDs as the bulk of the mass is below the detectable size limit of the OPC. As the geometric mean diameter increases in size or the width of the distribution becomes larger, a larger fraction of the particles enters the detectable range, slightly improving the results for the low-cost OPC. The high-end OPC is most able to adapt to the changes in the PSD due to its significantly smaller *D*_min_ (100 nm); there is roughly a 20 % difference across the entire range of PSDs shown. Unlike the low-cost OPC, a majority of the mass falls within the detectable range of the high-end OPC, resulting in little to no effect of changes to the PSD on the accuracy of the mass concentration measurement.

While previous work has highlighted the importance of the varying PSD and its effect on making accurate mass measurements with OPSs (Di Antonio et al., 2018; Gao et al., 2013; Malings et al., 2020), the effect of missing mass - the mass below the lowest size bin of an OPC - has received relatively little attention. The standard way to treat this missing mass is to empirically correct via regression analysis (Dacunto et al., 2015; Malings et al., 2020). While this can mitigate absolute errors, it requires the assumption that the PSD is constant in shape, varying only in magnitude. With particle loadings that are mostly below tens of micrograms per cubic meter (μg m^−3^) throughout the United States, this assumption is unlikely to be a large source of absolute error. However, if the same approach were used in highly polluted environments where sub-300 nm aerosol loadings can easily reach hundreds of micrograms per cubic meter (μg m^−3^) (Bhandari et al., 2020; Gani et al., 2019, 2020), changes in the PSD are likely to lead to large errors (in both an absolute and relative sense) in mass loading measurements. Overall, nephelometers and OPCs with high minimum size cutoffs are prone to substantial uncertainties as the underlying PSD changes, whereas for OPCs with low minimum size cutoffs this effect is relatively minor.

## 4 Implications and future work

In this work, we have laid out a framework for understanding the sensitivity of low-cost optical particle sensors to the various physical and optical properties of aerosols. We described a new Mie-theory-based software package (*opcsim)* for modeling the response of OPSs to various aerosols and demonstrated its use for better understanding the strengths and limitations of various low-cost particle sensors. We also used the model to investigate how various potential pitfalls (e.g., changes to environmental conditions, mismatches between calibration particles and particles being measured) may contribute to errors in mass concentration measurements. A summary of these results is given in Table 3.

Consistent with previous studies, our results suggest that relative humidity is a large source of uncertainty for all OPSs when the aerosol is hygroscopic and relative humidities are above the deliquescence point, typically around 75 %; additionally, the error introduced by relative humidity is highly sensitive to the aerosols’ affinity for water. This is correctable, at least to first order, limiting the impact of RH error on final results (Crilley et al., 2018; Di Antonio et al., 2018; Malings et al., 2020). We showed that the aerosol optical properties are most important for low-cost OPCs and of medium importance for high-end OPCs and nephelometers. This is especially relevant when the aerosol is strongly absorbing, as the amount of scattered light can make small particles undetectable with inexpensive optical detectors. If it were possible to measure some proxy for aerosol composition in real time, it would be possible to vastly reduce this error and improve the accuracy of mass measurements using OPSs for real-time data collection. Finally, we showed that the underlying particle size distribution is very important for the accuracy of low-cost OPCs and nephelometers, while being of low relative importance for high-end OPCs that can properly count and size particles at low sizes. The ability of a given OPC to measure small particles is found to be important, with marginal improvements leading to large gains in ability to accurately infer mass. Additionally, the choice of calibrant is found to be extremely important for both nephelometers and OPCs. Ensuring that OPSs are calibrated intelligently (i.e., using particles similar to the aerosol to be detected) can lead to significant improvements in expected performance. Finally, the bin boundary definitions for an OPC are also important, as defining them with large overlap in expected *C*_scat_ values can lead to significant bin misassignment and therefore inaccurate mass calculations.

Table 4 summarizes these results within the context of measurements of representative real-world aerosol types. It provides an overview of the potential errors associated with different types of optical particle sensors under various scenarios, with recommendations for the type of calibration particles that would minimize errors in PM_2.5_ mass measurements. Generally, in environments where small particles (< 300 nm) comprise a large percentage of the total mass, low-cost OPCs will be subject to considerable error. This will also be the case in environments with substantial levels of light-absorbing aerosol, such as wildfires or soot-heavy environments. (Sensor calibration using absorbing particles could help mitigate this effect, though this would introduce new errors when measuring non-absorbing aerosol.) In environments in which the underlying aerosol size distribution is variable (especially on short sub-hourly timescales), such as urban environments or evolving wildfire plumes, nephelometers and low-cost OPCs will struggle to keep up with the changes in the relationship between the total scattered light and mass loading, leading to large variance in the mass estimates.

The estimates and recommendations given in Table 4 are not intended to be comprehensive, but rather serve as a starting point for characterizing the strengths and limitations of low-cost OPSs using Mie theory (and specifically the *opcsim* software package). Additional *opcsim* simulations carried out across a range of sensor designs, calibrant particles, and measured particle types could provide more comprehensive and quantitative estimates of errors in measured particle sizes and mass loadings, including for individual sensors and individual use cases. Future improvements to *opcsim* could be made to allow for the simulation of more complex aerosols (e.g., externally mixed populations, other particle morphologies) or the inclusion of more complex bin-assignment algorithms; comparison with laboratory studies (in which *M*_m_*/M*_a_ is measured rather than just estimated) would also be useful. Additionally, colocated data with size-resolved measurements would allow for improved validation of the OPC component of this model. It is hoped that the Mie-theory-based approach described here will lead to an improved understanding of the errors associated with low-cost optical PM measurements, insight into calibration techniques that minimize such errors, and ultimately guidance into the design of new PM sensors for improved low-cost measurements of air quality and human exposure.

## Supplementary Material

Supplemen

## Figures and Tables

**Table 1.** Characteristics of a selection of commercially available low-cost optical particle counters and nephelometers.

Manufacturer	OPS type	Model	λ (nm)	Viewing angle (∅_1_,∅_2_)	No. of size bins
Alphasense, Ltd.	OPC	OPC-N2	658	(32.0°, 88.0°)	16 (0.38–17.5 μm)
Alphasense, Ltd.	OPC	OPC-N3	658	(32.0°, 88.0°)	24 (0.35–40.0 μm)
Particle Plus	OPC		785	(58.0°, 118.0°)	6 (0.3–10.0 μm)
NOAA/Handix	OPC	POPS	405	(38.0°, 142.0°)	16 (0.132–3.65 μm)
Plantower	Nephelometer	PMS5003	~ 650	?^1^	6(0.3–10+ μm)^2^
Sharp	Nephelometer (photometer)	GP2Y1010AUOF	870–980	?^1^	1(?)^3^
Shinyei	Nephelometer (photometer)	PPD42NS	870–980	?^1^	1 (> 1 μm)
Samyoung	Nephelometer (photometer)	DSM501A	870–980	?^1^	1 (> 1 μm)

1 Unknown; not provided in the manufacturer’s technical data sheet or the technical literature.

2 The PMS5003 reports six bins; however, these are not actual size bins, but rather software-computed results (He et al., 2020).

3 No size detection limit for the Sharp sensor is listed in the literature or in the manufacturer’s technical data sheet.

**Table 2.** Aerosol optical and chemical properties used in this work.

Aerosol type	Refractive index	Hygroscopicity parameter *κ*^6^	Density (g cm^−3^)
Urban^1^	1.525 + 0.020*j*	0.40	1.35
Background^2^	1.520 + 0.008*j*	0.25	1.45
Marine^3^	1.384 + 0.001*j*	1.10	2.16
Dust^4^	1.555 + 0.003*j*	0.03	2.60
Wildfire^5^	1.570 + 0.002*j*	0.10	1.58

1 Chen et al. (2019), Cheung et al. (2020), Hussein et al. (2004), Jurányi et al. (2013), Raut and Chazette (2007), Rissler et al. (2014), Shepherd et al. (2018), Wehner and Wiedensohler (2003).

2 Levoni et al. (1997), Wang et al. (2014), Yin et al. (2015).

3 Levoni et al. (1997), Ueda et al. (2016), Zieger et al. (2017).

4 Koehler et al. (2009), Petzold et al. (2009), Rocha-Lima et al. (2018).

5 Bougiatioti et al. (2016), Laing et al. (2016), McMeeking (2004), Shepherd et al. (2018).

6 Petters and Kreidenweis (2007).

**Table 3.** Effects of changing environmental and/or aerosol parameters on the relative error in measured mass loading by different OPS types.

Parameter changed	OPS type		
	Low-cost OPC	High-end OPC	Nephelometer
RH and hygroscopicity (Fig. 2)	Very high (20 %−200 %) for hygroscopic materials when RH > ~ 75 %		
Optical properties (Fig. 4)*	Very high (30 %−100 %)	Medium (20 %−60 %)	Medium (20 %−75 %)
Particle size distribution (Fig. 5)	Very high 60 %−90 %	Low < 20%	High 50 %−70 %

* Primarily a source of error when an OPS calibrated with non-absorbing particles measures absorbing particles (or vice versa).

**Table 4.** Summary of expected performance and recommendations for calibration materials in the use of low-cost optical particle sensors to measure the particulate mass loadings of different aerosol types.

			Sensor performance by OPS type*		
Aerosol type	Aerosol properties	Suggested calibrant	Low-cost OPC	High-end OPC	Nephelometer
Fossil-fuel combustion	Very small GMD, mostly non-hygroscopic, moderate absorbing RI (Bond et al., 2002; Bond and Bergstrom, 2006; Chang et al., 2004)	Calibrate with aerosols closer in RI, such as from combustion sources	Will perform poorly due to the small GMD and absorption component of the aerosol	Will perform moderately well, though it will miss ultrafine particles	Can perform moderately well if calibrated using appropriate materials
Wildfire	Varying PSD, moderate absorbing component of RI (Laing et al., 2016; McMeeking, 2004)	Calibrate with aerosol of similar optical properties and GMD (ideally biomass smoke)	Will likely undersize and underestimate mass due to the absorbing component of the aerosol; the GMD will change with proximity to the source, leading to changes in accuracy	Will perform moderately well, though it may mis-size the particles as the properties of the aerosol change as the plume evolves	Can perform well under certain circumstances; moderate error should be expected as the GMD of the wildfire plume evolves
Urban	Varying GMD, moderate hygroscopicity (Hussein et al., 2004; Wehner and Wieden-sohler, 2003)	Calibrate with NIST urban aerosol or collected urban aerosol	Performance depends on uniformity of sources; large errors will occur as aerosol source (and GMD) changes	Will perform moderately well to well, though it will miss ultrafine particles	Will perform moderately well if averaged over a long period of time to normalize the GMD
Dust	Large GMD, non-hygroscopic (Petzold et al., 2009; Rocha-Lima et al., 2018)	Calibrate with Arizona road dust or collected dust	Likely to perform well given the large particle sizes	Likely to perform well given the large particle sizes	Likely to perform well

* Based on properties of the aerosol only and not external environmental parameters (i.e., RH).
