# Supplementary material for: Assessing the accuracy of low-cost optical particle sensors using a physics-based approach
Source: Atmos Meas Tech. Author manuscript; Available in PMC 2021 Mar 26. (PMC7995643; doi:10.5194/amt-13-6343-2020)
Supplement: Supplemen [file NIHMS1662426-supplement-Supplemen.pdf]

Supplement of Atmos. Meas. Tech., 13, 6343–6355, 2020
https://doi.org/10.5194/amt-13-6343-2020-supplement
© Author(s) 2020. This work is distributed under
the Creative Commons Attribution 4.0 License.

Atmospheric Measurement Techniques
Open Access

Supplement of

Assessing the accuracy of low-cost optical particle sensors using a physics-based approach

David H. Hagan and Jesse H. Kroll

Correspondence to: David H. Hagan (david.hagan@quant-aq.com) and Jesse H. Kroll (jhkroll@mit.edu)

The copyright of individual parts of the supplement might differ from the CC BY 4.0 License.

Supplemental Information

Impact of viewing angle of scattered light collection

The viewing angle for a given OPC can strongly influence its ability to correctly count and size particles. Increasing the viewing angle typically leads to a larger fraction of the incident light being collected which can be helpful when inexpensive light detectors are being used. In addition to the total signal of the scattered light, it can also lower the influence of the Mie resonance by integrating over a wider degree. Figure S1 shows the impact that widening the viewing angle can have on an OPC with the same laser wavelength (658 nm) – the smallest viewing angle (blue) collects the least amount of light and shows the largest impact from the Mie resonance, whereas the red line ,which has a viewing angle similar to that of a nephelometer, shows the least impact from the Mie resonance and collects the most light.

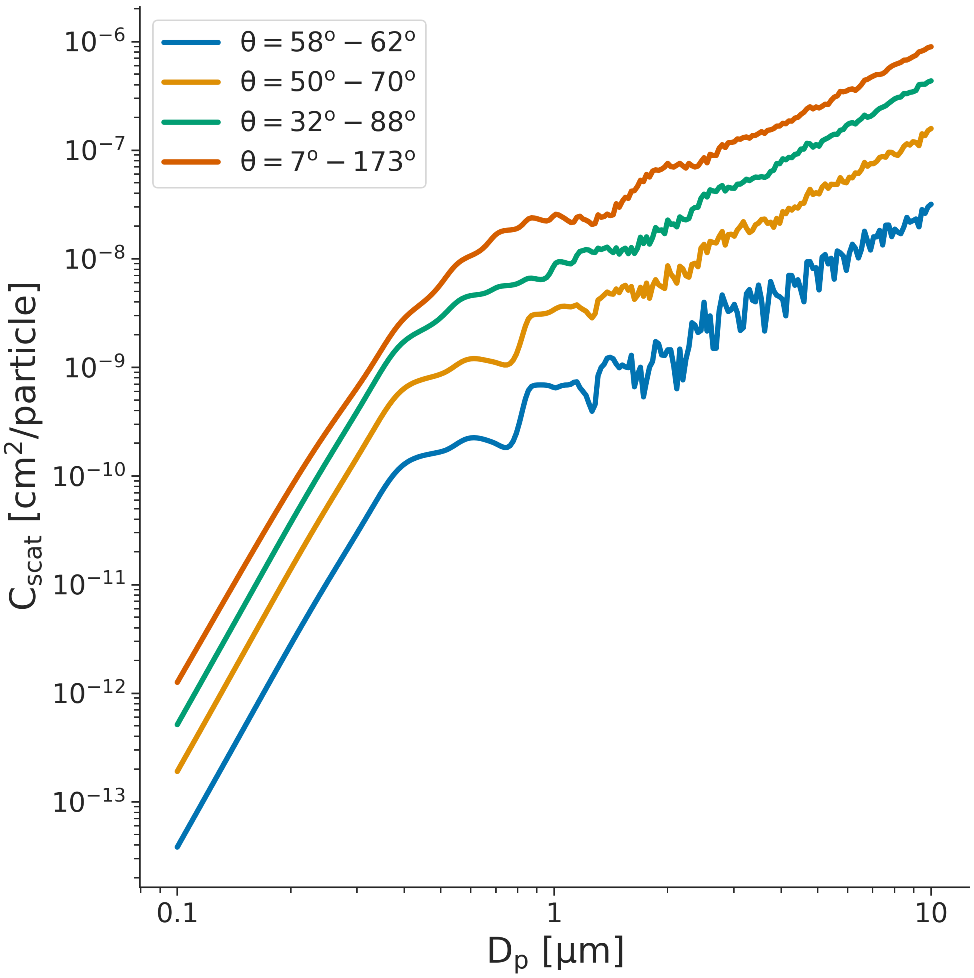

Figure S1. The amount of scattered light changes as a function of the OPC's viewing angle for a given incident laser wavelength. Above, the blue line depicts the total scattered light collected for a photometer-like instrument with a very narrow viewing angle. As the viewing angle is widened, the total amount of scattered light collected increases and the effect of the Mie resonance decreases.

Impact of changes in aerosol optical properties on OPC bin assignment

Here we investigate how bin assignment may be affected If the aerosol optical properties of the measured aerosol are different than the material used to calibrate an OPC. We compute the light scattering for a range of possible refractive index values and plot them in Figure S2 below for a 600 nm particle. Across a wide range of optical properties, the particle can be placed in 3 different bins or left out completely ("NO BIN"). However, it should be noted that fairly large changes in either the real or imaginary part of the RI need to change in order for a particle to be assigned to a different bin.

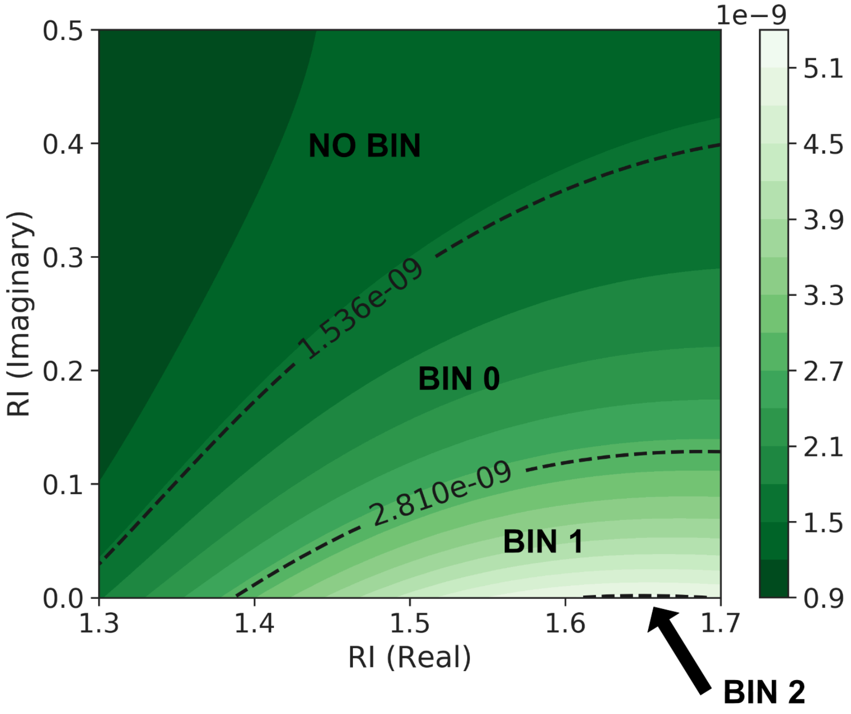

Figure S2. Bin assignment as a function of refractive index for a 600 nm particle. An OPC with 16 bins between 380 nm and 17.5 µm and a 658 nm laser calibrated using PSLs was used to generate this data. Data are colored by the computed integrated scattering cross section.

If we complete the same exercise for a 1 µm particle, the results are quite different. Small changes in the absorbing component of the refractive index can lead to large differences in how the particle is assigned to a bin. Over the entire range of optical properties shown in Figure S3, the 1µm particle can be assigned to 5 different bins (or NO BIN). For this specific OPC, this means the 1 µm particle can be assigned a diameter of anywhere from 0 µm to 1.26 µm. This indicates that for large particles, differences in optical properties of calibration aerosol and measured aerosol can lead to large errors in bin assignments (and hence mass measurements).

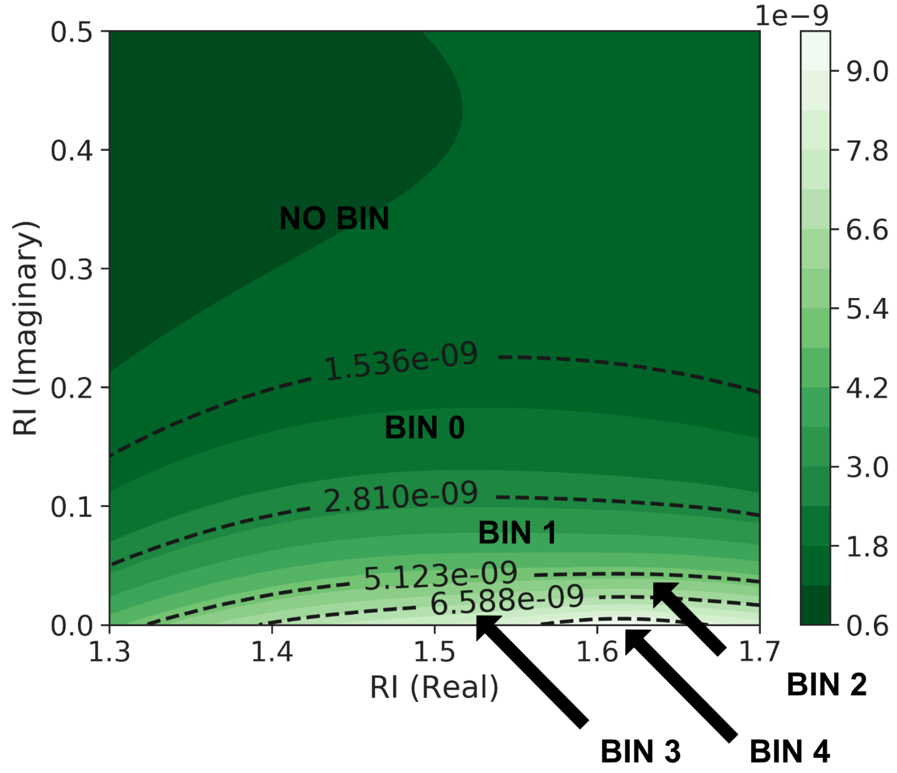

Figure S3. Bin assignment as a function of refractive index for a 600 nm particle. An OPC with 16 bins between 380 nm and 17.5 μm and a 658 nm laser calibrated using PSLs was used to generate this data. Data are colored by the computed integrated scattering cross section.

Impact of scattering angle have on nephelometer response

Here we investigate how the range of angles over which scattered light is collected affects measurements by nephelometers. To determine this effect, we computed the response factor for three nephelometers with scattering angles of (1) 7°-173°; (2) 45°-135°; and (3) 40°-50°, for a range of particle size distributions (as in Figure 5). While the response factors do indeed depend on viewing angle, all results are qualitatively similar, and so the results in Figure 5 are not strongly dependent on viewing angle.

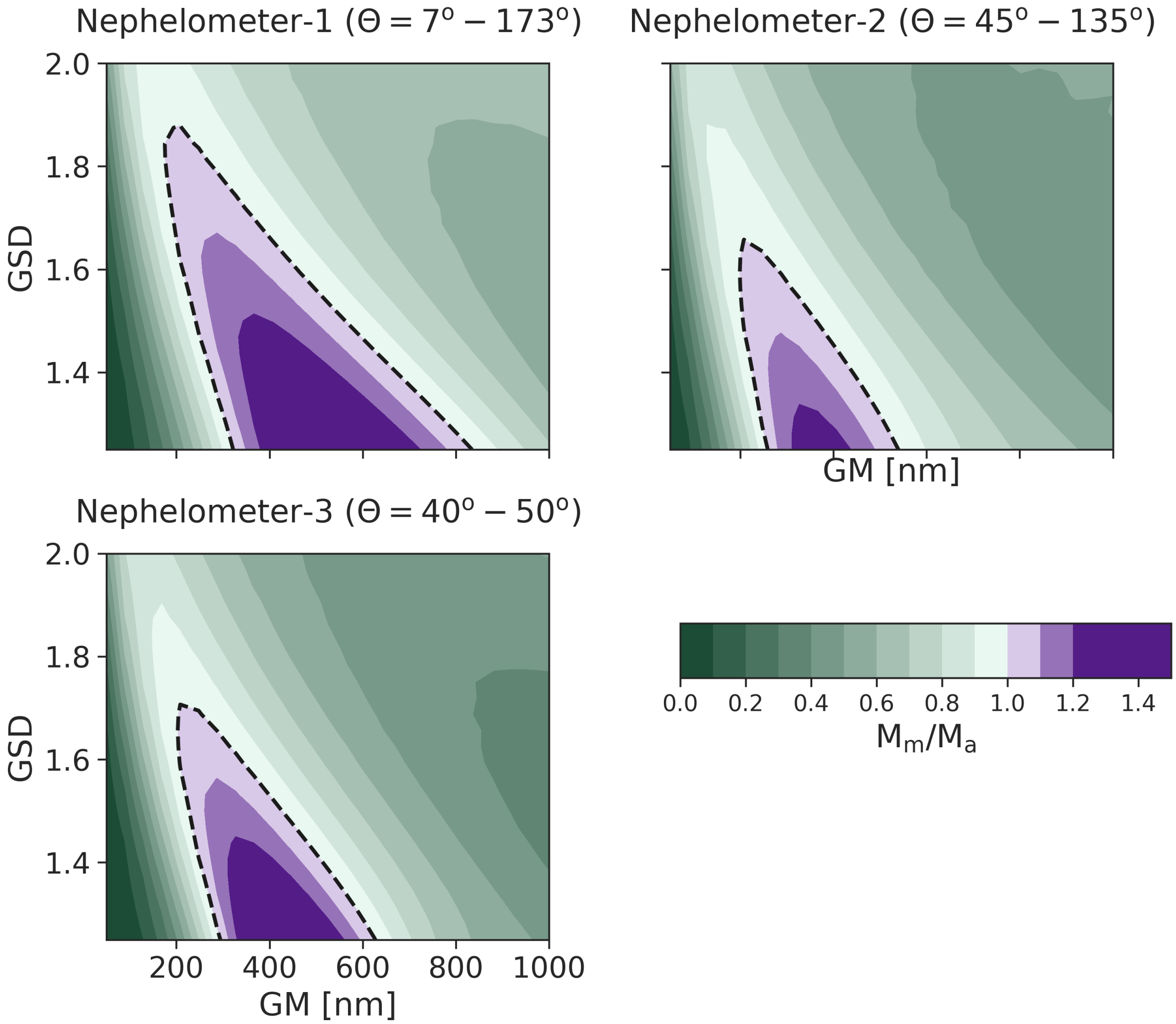

Figure S4. Effect of viewing angle range on nephelometer response. As in Figure 5, all nephelometers were calibrated using ammonium sulfate and all measured particles are ammonium sulfate, with a range of GMs and GSDs. Absolute mass accuracy (M_m/M_a) differs in each case, but the results are qualitatively similar across all angle ranges,
